# New Advances in Neuroimmunology and Neuroinflammation

**DOI:** 10.3390/brainsci16050464

**Published:** 2026-04-26

**Authors:** Junhui Wang

**Affiliations:** 1 Thyropathy Hospital, Sun Simiao Hospital, Beijing University of Chinese Medicine, Tongchuan 727000, China; junhui@lunenfeld.ca; 2 Lunenfeld-Tanenbaum Research Institute, Mount Sinai Hospital, Toronto, ON M5G 1X5, Canada

The field of neuroimmunology has changed dramatically over the last ten years. The two systems that were thought to be completely separate have now been found to have a very complex interaction, and this has become a basis for understanding brain function, diseases, and healing [1,2]. Neuroinflammation, which was originally considered to be just a side effect of brain injury, is now recognized as a process that can result in either the protection or the harming of brain tissue via a range of neurological and psychiatric diseases such as multiple sclerosis, Alzheimer’s, depression, and schizophrenia [3,4,5].

Even with these advancements, there is still a lot of work to be done. How exactly cells of the peripheral immune system interact with the brain and spinal cord, to what extent the different types of microglia affect the outcome of the disease, and ways of devising therapies that alter neuroimmune interactions without reducing immune system capabilities are just some of the topics that we need to study further. These issues become even more complex when considering the recent findings of sex differences in neuroimmune responses and the impact of the gut–brain axis on neural inflammation.

The current Special Issue, titled “**New Advances in Neuroimmunology and Neuroinflammation**” consists of nine papers that together shed light on these problems. The studies cover the entire spectrum, from the basic science research of the signaling pathways activated by cytokines to the clinical observation of the relationship between the peripheral immune markers and neurological phenotypes. By compiling different research methods, experimental models, neuroimaging, and translational clinical studies, we intended to produce a reference that unites the usual division between preclinical neuroscience and clinical neurology.

Milligan et al. [6] found that IL-6 trans-signaling is a major factor that drives the progression of amyotrophic lateral sclerosis (ALS). About 60% of ALS patients carry the IL6R Asp358Ala mutation, which results in increased levels of soluble IL-6 receptor (sIL-6R) and thereby promoting trans-signaling. In the SOD1 G93A mouse model, enhanced trans-signaling leads to a more rapid progression of symptoms and pathological changes, indicating that targeting IL-6 trans-signaling could be a beneficial approach for modifying the disease [6]. Alonge et al. [7] pointed out that TREM2 is a gatekeeper for microglial activation and the formation of disease-associated microglia (DAM). Their review sheds light on the role of TREM2 as a transmembrane protein on myeloid cells. Animal experiments show that lack of TREM2 exacerbates α-synuclein-induced inflammatory responses in the brain, which in turn leads to the loss of dopaminergic neurons [7]. On top of that, there is evidence that higher amounts of soluble TREM2 (sTREM2) in the cerebrospinal fluid of the PD patients serve as a marker for the risk of cognitive decline [7]. In ALS, microglia adopt to DAM in response to both intrinsic and extrinsic cues, thereby contributing to inflammation and neuronal injury. In this DAM model, microglia transition from a homeostatic state to stage 1 (TREM2-independent) and subsequently to stage 2 (TREM2-dependent). The initial transition from homeostatic microglia to DAM is triggered by neuronal injury and occurs independently of TREM2. This stage is associated with responses aimed at cellular repair and a relatively restrained or initially anti-inflammatory phenotype. The second stage depends on TREM2 activation and is characterized by the upregulation of phagocytic pathways and lipid metabolism genes, including CD9, Lpl, Cst7, and ApoE [8]. In Alzheimer’s disease (AD), DAM emerges in response to amyloid-β accumulation and neuronal injury, transitioning from a homeostatic to an activated, disease-specific state. Meanwhile, dysfunction of the blood–brain barrier permits the entry of peripheral immune signals and disrupts brain homeostasis, which further enhances DAM activation and amplifies neuroinflammatory processes [9]. The role of microglia in the pathogenesis of AD is receiving increased attention. Hansen et al. [10] discussed the concept of how microglia activate the NLRP3 inflammasome to perpetuate neuroinflammatory circuits. Meanwhile, Matejuk et al. [11] explored the different components of neuroinflammation in AD such as astrocytes–microglia interactions. The most recent research has presented the idea of the “neuroimmune axis”, which attends to the interplay between the peripheral immune system and the central nervous system (CNS) in Alzheimer’s disease. It was demonstrated [12] that the gut microbiome through the gut–brain axis influences microglial function, therefore resulting in a “double-edged sword” relationship in AD, because they can either provide neuroprotection or mediate pathological damage. To sum up, neuroimmune mechanisms are of primary importance in the pathophysiology of neurodegenerative diseases. Blocking IL-6 trans-signaling, targeting TREM2, the hypoxia-inflammation cycle, and purinergic pathways may offer new avenues for the treatment of these disorders. Microglia are the main immune cells of the CNS, and their activation state determines whether there is neuroprotection or neurodegeneration [10,12]. On the other hand, these mechanisms’ complex interrelations remain largely unknown. Upcoming studies should prioritize the following: first, developing means to switch microglia to a brain-protective phenotype; second, unraveling the contributions of the neuroimmune axis in the disease course; third, moving neuroinflammation-targeted therapies from bench to bedside.

We hope this collection will inspire many to ask more questions and to break down the barriers between disciplines together. Uncovering the answers to these remaining questions will be a challenge beyond the capabilities of a single laboratory or specialty working in isolation. We believe that neuroimmunology in the next decade will not be centered around the outstanding discoveries of individual teams, but the networks they build.

## Data Availability

Not applicable.
